# Oral Lichen Planus: Current Evidence, Clinical Challenges, and the Search for Optimal Management

**DOI:** 10.3390/jcm15207995

**Published:** 2026-10-15

**Authors:** Masanobu Abe

**Affiliations:** Department of Dentistry & Oral Surgery, The Cancer Institute Hospital of Japanese Foundation for Cancer Research, 3-8-31 Ariake, Koto, Tokyo 135-8550, Japan; masanobu.abe@jfcr.or.jp; Tel.: +81-3-3520-0111; Fax: +81-3-3520-0141

**Keywords:** oral lichen planus, oral potentially malignant disorders, diagnosis, management, malignant transformation

## Abstract

Oral lichen planus (OLP) is a chronic, immune-mediated inflammatory disease of the oral mucosa with an incompletely understood etiology and a diverse clinical presentation. Because OLP is often refractory and follows a chronic or relapsing course, accurate diagnosis and appropriate long-term management remain important clinical challenges. The diagnosis of OLP is based on clinical findings and histopathological examination, with consideration of a variety of conditions that may mimic OLP, including oral lichenoid lesions, autoimmune blistering diseases, candidiasis, and leukoplakia. Management is primarily aimed at controlling inflammation and alleviating symptoms. Elimination of precipitating or aggravating factors, improvement of oral hygiene, and appropriate dental treatment are important components of long-term disease management. Topical corticosteroids remain the mainstay of local therapy, while topical calcineurin inhibitors, such as tacrolimus and pimecrolimus, may provide useful alternatives, particularly in steroid-refractory cases. Laser therapy may also be considered as an adjunctive treatment in selected patients. Systemic corticosteroids are generally reserved for severe, extensive, or refractory disease. In addition, various adjunctive and alternative therapies, including herbal medicines, phytochemicals, vitamins, micronutrients, hydroxychloroquine, and methotrexate, have been investigated. Although several of these approaches have shown promising therapeutic effects, the available evidence remains limited and further high-quality clinical studies are required. OLP is currently classified among the oral potentially malignant disorders, although the magnitude of its malignant transformation risk remains debated. Therefore, long-term surveillance is important because some patients may develop oral squamous cell carcinoma. At present, the management of OLP focuses primarily on symptom relief and disease control rather than cure. A comprehensive and individualized approach that integrates accurate diagnosis, elimination of aggravating factors, oral health management, appropriate selection of topical and systemic therapies, and long-term surveillance is therefore important. Further clarification of the underlying mechanisms of OLP and well-designed clinical studies may contribute to more effective and individualized approaches to its diagnosis and management.

## 1. Introduction

Oral lichen planus (OLP) is a chronic, immune-mediated inflammatory disease of the oral mucosa with an incompletely understood etiology and pathogenesis [1]. It exhibits a wide variety of clinical manifestations, ranging from asymptomatic reticular white lesions to erythematous, erosive, and ulcerative lesions associated with pain and burning sensations [1]. Because these clinical features may change over time and overlap with those of other oral mucosal diseases, including oral lichenoid lesions, autoimmune blistering diseases, candidiasis, and leukoplakia, accurate diagnosis requires careful clinical evaluation and, when appropriate, histopathological examination [1,2].

OLP is often refractory and follows a chronic or relapsing course, making long-term disease management a clinical challenge [1]. Although elimination of precipitating or aggravating factors, improvement of oral hygiene, and appropriate dental treatment may contribute to symptom control, topical corticosteroids remain the mainstay of treatment [1,3]. However, some patients respond inadequately or experience repeated relapse, and various topical, systemic, and adjunctive therapies have therefore been investigated. These include topical calcineurin inhibitors, laser therapy, systemic immunomodulatory agents, herbal medicines and phytochemicals, vitamins and micronutrients, and other approaches [3,4]. Although some of these treatments have shown promising results, their efficacy and clinical applicability remain variable, and no definitive cure for OLP has yet been established [3,4].

Another important consideration in the long-term management of OLP is its potential for malignant transformation. OLP is regarded as an oral potentially malignant disorder, although the magnitude of its malignant transformation risk remains a matter of debate. Despite the relatively low reported risk of transformation to oral squamous cell carcinoma, careful diagnosis and long-term surveillance remain essential [5].

Thus, the management of OLP requires more than suppression of local inflammation and symptoms. It involves accurate diagnosis, identification and elimination of precipitating or aggravating factors, maintenance of oral health, individualized selection of treatment, and appropriate long-term surveillance [1,3,5]. This review summarizes current perspectives on the diagnosis and management of OLP, with particular emphasis on conventional and emerging therapeutic approaches and the potential for malignant transformation. By integrating these aspects, we aim to provide a practical overview of the current challenges in OLP management and to highlight potential directions for future research.

## 2. Pathology and Pathogenesis of Oral Lichen Planus

Lichen planus is a chronic, immune-mediated inflammatory disease of unclear etiology that primarily affects the skin and mucosal surfaces [1,6,7]. Lichen planus involving the oral mucosa is referred to as oral lichen planus (OLP) [8,9,10], which typically presents as fine reticulated white lines known as Wickham’s striae on the bilateral buccal mucosa, often with surrounding erythema [8,10,11]. However, the clinical presentation of OLP is diverse. In addition to white lesions, erythema, erosions, ulcers, and, less commonly, blisters may occur, and the clinical appearance may change over time [8,10,12]. The lesions in OLP are not limited to the buccal mucosa and may involve the buccal transition area, gingiva, tongue margin, dorsum of the tongue, lips, and/or corners of the mouth [8,10].

Some patients with OLP also have lichen planus involving other mucosal or cutaneous sites [1,8,13]. The reported prevalence of OLP varies among studies, partly because of differences in diagnostic criteria and study populations. OLP is more commonly reported in women and typically presents during middle age [1,8,10]. Histopathologically, OLP is characterized by basal cell liquefaction, a band-like subepithelial infiltrate predominantly composed of lymphocytes, and varying degrees of epithelial hyperkeratosis. Saw-toothed rete ridges and Civatte bodies may also be observed. Although these features are not individually pathognomonic, their combination supports the diagnosis of OLP when correlated with the clinical findings [14,15].

Although the pathogenesis of OLP is not fully understood, it is generally considered to involve a predominantly cell-mediated immune response [1,13,16]. The activation and recruitment of T lymphocytes, together with the release of inflammatory cytokines and chemokines, contribute to epithelial injury and perpetuation of the inflammatory response [1,13,16]. These immune-mediated epithelial injuries contribute to the development of the characteristic hyperkeratotic, erythematous, and erosive lesions of OLP [1,13].

Recent studies have suggested that the immunopathogenesis of OLP involves a more complex imbalance among T-cell subsets than previously recognized. In particular, increasing evidence implicates the Th17/Treg axis in the pathogenesis of OLP. Th17 cells and their signature cytokine IL-17 are increased in the lesional tissues and peripheral blood of patients with OLP, and IL-17 expression appears to be more pronounced in erosive than in reticular OLP [17]. Conversely, impaired regulatory T-cell function may contribute to insufficient control of local inflammation, resulting in an imbalance between proinflammatory Th17 responses and immunoregulatory Treg responses [17,18]. Immunohistochemical studies have further demonstrated the presence of FoxP3+ Treg cells in OLP lesions. In a comparative study of OLP and oral epithelial dysplasia with lichenoid features, FoxP3+ Treg cells were detected in OLP tissues and were more prominent in areas with higher tertiary lymphoid structure scores, suggesting a relationship between local lymphoid organization and Treg infiltration [19]. More recently, tissue-based analyses using multiplex immunofluorescence and single-cell RNA sequencing identified Treg populations within OLP lesions and demonstrated reduced expression of immunosuppressive molecules, suggesting impaired regulatory activity despite the presence of Tregs [20]. Experimental evidence further suggests that microbial stimulation can influence this balance. For example, Escherichia coli was shown to activate the TLR4/NF-κB pathway in oral keratinocytes and OLP-derived T cells, increasing IL-6, IL-17, and CCL20 expression and promoting a Th17/Treg imbalance [18]. These findings suggest that dysregulation of the Th17/Treg axis may contribute to the persistence and amplification of chronic inflammation in OLP.

The JAK/STAT signaling pathway has also emerged as a potential molecular pathway linking cytokine signaling to epithelial and immune-cell responses in OLP. A recent immunohistochemical study demonstrated increased expression of JAK1 and phosphorylated JAK1 in both epithelial cells and inflammatory infiltrates in OLP compared with normal oral mucosa, supporting activation of JAK-mediated signaling in OLP lesions [21]. Furthermore, a 2026 study suggested that JAK2/STAT3 signaling in oral keratinocytes is involved in the homing of intraepithelial lymphocytes and the maintenance of mucosal barrier integrity [22]. These findings provide a rationale for further investigation of JAK/STAT signaling as a potential therapeutic target in OLP, although clinical evidence supporting JAK/STAT-targeted therapy is currently lacking.

Epithelial barrier dysfunction may represent another important component of OLP pathogenesis. In addition to immune-mediated keratinocyte injury, abnormalities in epithelial cell–cell adhesion and tight junctions may increase mucosal permeability and facilitate interactions between luminal antigens and the underlying immune system. Reduced expression of E-cadherin and several tight-junction proteins, including claudin-1, claudin-4, occludin, and zonula occludens-1, has been demonstrated in OLP lesions, with reduced occludin expression associated with greater disease severity [23]. These findings raise the possibility that impaired epithelial barrier function and immune dysregulation may form a reciprocal cycle in which epithelial damage facilitates antigen exposure and immune activation, while persistent inflammation further compromises epithelial integrity [22,23].

The oral and gut microbiome may also participate in this complex pathogenic network. Recent studies using 16S rRNA sequencing and multi-omics approaches have demonstrated differences in the composition of the oral microbiome between patients with OLP and healthy controls, as well as between erosive and non-erosive OLP [24,25]. Although these findings do not establish a causal relationship, experimental studies suggest that specific microbial stimuli can modulate inflammatory signaling and the Th17/Treg balance [18]. Moreover, a recent study reported gut microbiome dysbiosis in patients with OLP, including a reduction in short-chain fatty acid-producing bacteria, which was associated with impaired Treg activation [20]. Thus, emerging evidence suggests that interactions among the microbiome, epithelial barrier, and mucosal immune system may contribute to the initiation and persistence of OLP. However, whether microbiome alterations represent a cause, a consequence, or a disease-modifying factor in OLP remains to be determined.

Multiple factors are thought to be involved in the pathogenesis of OLP [1,8,13,26,27]. Genetic predisposition has been suggested to contribute to susceptibility to lichen planus, including associations with specific human leukocyte antigens (HLAs) [28]. Familial lichen planus has been reported to occur at a younger age and to show a more widespread clinical presentation and a tendency toward relapse compared with nonfamilial disease [29]. Familial OLP is rare, but familial clustering and multigenerational occurrence support a possible genetic contribution to its pathogenesis [30,31]. Acquired factors, including environmental and systemic factors, may also contribute to the development and exacerbation of OLP. An association between OLP and hepatitis C virus (HCV) infection has been reported, although the strength of this association varies among populations and geographic regions [32,33,34]. Medications, including NSAIDs and antihypertensive agents, have been implicated as potential triggers of oral lichenoid reactions [35]. In addition, systemic conditions, oral conditions, and lifestyle or environmental factors have been associated with the development and/or exacerbation of OLP [8,36,37,38,39] (Table 1).

## 3. Diagnosis of Oral Lichen Planus

The diagnosis of OLP relies on careful clinical evaluation and, when indicated, histopathological examination of a biopsy, with clinicopathological correlation being particularly important in diagnostically challenging cases [1,2,10,14,47]. The differential diagnosis of OLP includes a broad range of oral mucosal disorders, including oral leukoplakia, autoimmune blistering diseases, chronic ulcerative stomatitis, candidiasis, systemic lupus erythematosus, and oral lichenoid lesions [2,10,14,47]. Mucous membrane pemphigoid (MMP) may clinically resemble erosive OLP, particularly when gingival lesions are present, and histopathological examination with direct immunofluorescence is useful for distinguishing these conditions [48]. Lichen planus pemphigoides (LPP), although rare, may also show clinical and histopathological features overlapping with OLP and requires careful clinicopathological evaluation [49,50]. Chronic ulcerative stomatitis should also be considered in patients with persistent painful oral erosions or ulcers, particularly when the clinical presentation resembles erosive OLP [51,52,53]. Oral candidiasis may mimic or coexist with erosive OLP and should therefore be considered in the differential diagnosis, especially in patients receiving topical corticosteroid therapy [44].

Among these differential diagnoses, oral lichenoid lesions (OLLs), including oral lichenoid contact lesions (OLCLs), oral lichenoid drug reactions (OLDRs), and lichenoid lesions associated with graft-versus-host disease (OLL-GVHD), are of particular clinical importance because their clinical and histopathological features may substantially overlap with those of OLP [40,41,42,43,54,55,56,57]. Distinguishing OLP from these OLLs can therefore be challenging, and the optimal diagnostic criteria and clinicopathological distinctions between these entities have yet to be fully established [2,10,43,55]. Unlike OLP, OLLs may be associated with identifiable triggering factors, such as contact allergens, medications, or systemic disease [40,41,42,43,54,55,56,57]. However, considerable clinical and histopathological overlap exists, and no single histopathological feature reliably distinguishes OLP from all forms of OLL [14,40,55]. A diagnosis of OLP should therefore not be made solely on the basis of histopathological findings; the distribution and morphology of lesions, symmetry, presence of cutaneous or other mucosal lesions, medication history, exposure to potential contact allergens, and relevant systemic conditions should also be considered.

An additional diagnostic challenge is the presence of epithelial dysplasia in lesions clinically and histologically resembling OLP. The relationship between OLP and epithelial dysplasia remains controversial. Dysplastic epithelial changes may occasionally be identified in lesions diagnosed as OLP, but it is uncertain whether such lesions represent OLP with secondary dysplasia, a lichenoid lesion with dysplasia, or another potentially malignant disorder accompanied by lichenoid inflammation. Moreover, some reactive epithelial changes associated with lichenoid inflammation may mimic dysplasia histologically, making the distinction difficult [55,58]. Therefore, when epithelial dysplasia is identified in an OLP-like lesion, the diagnosis should be based on careful clinicopathological correlation rather than on the presence of lichenoid inflammation alone. This distinction is particularly important when assessing malignant transformation risk, because studies that combine OLP with OLLs or include lesions with epithelial dysplasia may produce substantially different estimates of malignant transformation [5,55,58,59].

## 4. Current Therapy for Oral Lichen Planus

Oral lichen planus (OLP) is generally refractory and often follows a chronic or relapsing course, requiring individualized long-term management. The primary goals of treatment are to reduce pain and other symptoms, suppress clinically significant inflammation, promote healing of erosive or ulcerative lesions, and maintain oral function and quality of life rather than to achieve definitive cure. Treatment is broadly divided into local and systemic therapy. Topical corticosteroids remain the mainstay of treatment for symptomatic OLP, particularly for erythematous, erosive, and ulcerative disease [4,60,61,62]. When precipitating or aggravating factors are suspected, their elimination may contribute to symptomatic improvement. Appropriate oral hygiene and dental treatment are also important components of long-term disease management [45,46]. If local therapy fails to adequately control the disease, escalation to other topical or systemic therapies may be considered according to disease severity, extent, clinical subtype, comorbidities, and response to treatment.

**Figure 1:**
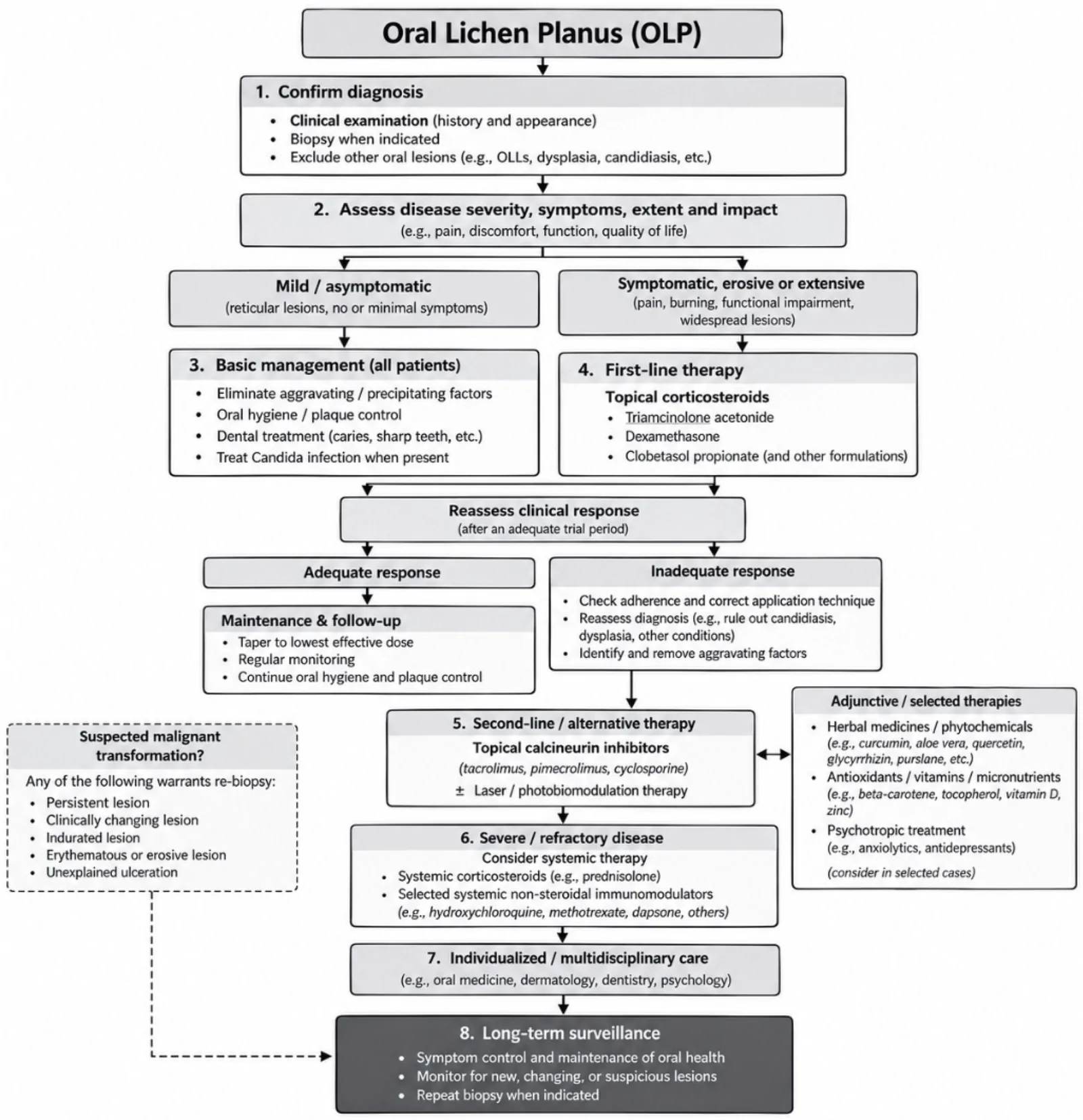
Treatment algorithm for oral lichen planus.

Because the available evidence is heterogeneous and many clinical trials are small, the evidence base for OLP treatment should be interpreted cautiously. Systematic reviews and meta-analyses have generally supported the efficacy of topical corticosteroids and calcineurin inhibitors, but the certainty of evidence is frequently low or very low because of small sample sizes, methodological limitations, heterogeneity of interventions and outcome measures, and short follow-up periods [3,4]. Accordingly, the therapeutic hierarchy proposed below reflects the current balance between efficacy, safety, and clinical experience rather than formal guideline-based GRADE recommendations. A stepwise treatment algorithm incorporating these therapeutic considerations is presented in Figure 1.

A stepwise approach to the management of oral lichen planus (OLP), incorporating basic oral care, topical corticosteroids as first-line therapy, treatment escalation for refractory or severe disease, selected adjunctive therapies, and long-term surveillance. Treatment should be individualized according to disease severity, symptoms, treatment response, and patient-specific factors. Persistent, clinically changing, or otherwise suspicious lesions should be reassessed and biopsied when clinically indicated.

## 5. Local Therapy for OLP

The evidence supporting local therapies for OLP is heterogeneous. Topical corticosteroids remain the first-line local pharmacological treatment for symptomatic OLP, whereas other approaches have been investigated mainly as adjunctive or alternative therapies, with varying levels of supporting evidence. When precipitating or aggravating factors are suspected, their elimination may improve or relieve symptoms. Candida infection should be identified and treated when present, particularly in patients receiving topical corticosteroids [36,44].

### 5.1. Topical Corticosteroids

Topical corticosteroids are the most widely used and best-established treatment for symptomatic OLP because of their anti-inflammatory effects [4,60,61,62]. Triamcinolone acetonide, dexamethasone, and clobetasol propionate are commonly used, with the choice of potency and formulation tailored to lesion severity, extent, and anatomical location [60,61,62]. Other formulations, including beclomethasone propionate spray and triamcinolone acetonide patches, may be considered according to lesion location and the patient’s ability to apply the medication.

A systematic review and network meta-analysis of randomized clinical trials demonstrated clinical benefits of topical corticosteroids among treatments for OLP [4]. The Cochrane review specifically evaluating corticosteroid therapies found low-certainty evidence that topical corticosteroids may improve pain compared with placebo, although the certainty of evidence for other clinical outcomes was limited [61].

*Overall evidence: Low to very low, largely because of methodological limitations, heterogeneity, and small sample sizes of the available clinical studies.*

*Clinical position: Established first-line local pharmacological treatment for symptomatic OLP.*

Thus, topical corticosteroids should remain the standard first-line pharmacological treatment, particularly for symptomatic erosive or ulcerative disease. Their principal advantages are established clinical experience, relatively predictable efficacy, local administration, and avoidance of systemic immunosuppression. Their principal limitations are secondary candidiasis, mucosal irritation, and the practical difficulties of prolonged treatment [36,44]. Long-term treatment should therefore be periodically reassessed, with dose and frequency adjusted according to clinical response.

### 5.2. Topical Calcineurin Inhibitors

Topical calcineurin inhibitors (TCIs), particularly tacrolimus and pimecrolimus, are the most extensively studied alternatives to topical corticosteroids. Topical cyclosporine has also been investigated in OLP, mainly in small randomized or controlled clinical trials [63,64,65]. Although an early double-blind trial reported significant clinical improvement with topical cyclosporine compared with vehicle [64], subsequent controlled trials found no clear advantage over topical corticosteroids [63,65]. Thus, the overall evidence remains limited and inconsistent.

A systematic review and meta-analysis found no significant difference between topical tacrolimus and corticosteroids in clinical resolution, although the available evidence remains limited by methodological heterogeneity and small sample sizes [66]. Another systematic review and meta-analysis similarly found broadly comparable efficacy between topical calcineurin inhibitors and topical corticosteroids [67]. Individual randomized trials have reported efficacy of tacrolimus comparable with clobetasol and of pimecrolimus comparable with tacrolimus in steroid-refractory erosive OLP [68,69].

*Overall evidence: Low to moderate, particularly for short-term efficacy.*

*Clinical position: Conditional second-line treatment, particularly for corticosteroid-refractory disease or when prolonged corticosteroid use is undesirable.*

From an efficacy–safety perspective, TCIs offer the advantage of avoiding corticosteroid-associated local adverse effects during prolonged treatment, but they are not clearly more effective than topical corticosteroids. Burning or irritation at the application site is relatively common, and the long-term safety of TCIs in OLP remains incompletely established. Importantly, reports of malignant transformation during TCI treatment cannot establish a causal relationship because OLP itself carries a potential risk of malignant transformation [5]. Therefore, TCIs should be regarded as an alternative rather than a replacement for topical corticosteroids.

Overall, current evidence supports a treatment hierarchy in which topical corticosteroids remain the preferred first-line treatment, whereas TCIs are reasonable second-line agents for selected patients. This interpretation is also consistent with clinical practice recommendations in which TCIs are suggested for OLP that is refractory to topical corticosteroids [66,67,68].

### 5.3. Plaque Control and Adjunctive Oral Care

Plaque control is particularly relevant in patients with gingival OLP or desquamative gingivitis. A systematic review found that plaque control may improve clinical outcomes in OLP with gingival manifestations, while structured plaque control has been reported to modify inflammatory biomarkers [45,46]. Accordingly, gentle and effective oral hygiene should be encouraged, particularly in patients with erosive or ulcerative lesions. Comprehensive dental care is important for maintaining oral health and minimizing local sources of irritation and inflammation. However, the available evidence is limited, particularly regarding its effects on disease activity beyond improvement of local plaque-related inflammation.

Mouthwashes may be used as adjunctive treatment for symptom relief and oral hygiene; however, current evidence is insufficient to establish any specific mouthwash as a disease-modifying treatment for OLP.

### 5.4. Laser and Photobiomodulation Therapy

Laser-based therapies, including photobiomodulation, and high-intensity laser therapy, have been investigated as adjunctive approaches for symptomatic or refractory OLP [70,71]. Recent systematic review and meta-analysis data suggest potential improvements in clinical symptoms and signs; however, treatment protocols, laser parameters, follow-up periods, and outcome measures vary considerably among studies [70].

*Overall evidence: Low.*

*Clinical position: Adjunctive treatment option for selected patients, particularly when conventional topical therapy is inadequate or poorly tolerated.*

The principal potential advantage of laser-based therapy is the possibility of reducing pain and inflammatory activity without additional pharmacological exposure. However, the absence of standardized treatment protocols and the limited number of high-quality comparative trials prevent laser therapy from replacing established pharmacological treatment. Thus, it should currently be considered an adjunctive rather than first-line treatment.

### 5.5. Herbal Medicines and Traditional Medicine

Herbal medicines and traditional Chinese medicine have attracted considerable interest as complementary treatments for OLP. A systematic review published in 2020 suggested potential benefits of herbal interventions, but emphasized the methodological limitations and heterogeneity of the available studies [72]. More recently, a systematic review of 19 randomized controlled trials involving 1164 participants found that combinations of Chinese patent medicines and corticosteroids were associated with improvements in several clinical outcomes compared with corticosteroids alone [73]. However, the authors highlighted concerns regarding study quality and heterogeneity. A network meta-analysis similarly suggested potential benefits of traditional Chinese medicine-based approaches, but the findings remain difficult to interpret because of heterogeneity and limitations of the underlying studies [74].

*Overall evidence: Low to very low.*

*Clinical position: Adjunctive or investigational treatment; routine use cannot currently be recommended.*

An important limitation of this evidence is that many studies originate from single regions, use heterogeneous herbal formulations, and differ substantially in diagnostic criteria, treatment regimens, outcome measures, and follow-up periods. Consequently, favorable results from individual trials cannot readily be generalized to all herbal preparations or traditional medicine approaches. In addition, standardization of active ingredients, pharmacokinetic data, drug interactions, and long-term safety are insufficiently established for many preparations. Therefore, herbal medicines should currently be regarded as investigational or adjunctive therapies rather than evidence-equivalent alternatives to topical corticosteroids [72,73,74,75].

### 5.6. Antioxidants, Vitamins, and Micronutrients

Antioxidant therapy has also been investigated as a complementary treatment for OLP. A systematic review and meta-analysis including 19 studies reported improvements in pain and clinical scores, but substantial heterogeneity among studies substantially limits the certainty of these findings [76]. Individual studies have examined beta-carotene, tocopherol, curcumin, and related interventions. Beta-carotene supplementation reduced micronuclei in exfoliated oral epithelial cells, whereas topical tocopherol improved lesion surface area without demonstrating a significant reduction in pain compared with placebo [77,78]. A randomized controlled trial also reported greater clinical improvement when nanocurcumin was combined with topical triamcinolone than with triamcinolone alone [79].

*Overall evidence: Low.*

*Clinical position: Adjunctive treatment option in selected patients; routine use is not currently supported by sufficient evidence.*

Vitamin D has similarly been investigated as a potential adjunctive treatment. A systematic review suggested possible therapeutic potential, while subsequent studies have reported associations between vitamin D status and OLP and possible clinical benefits of supplementation [80,81,82]. However, the optimal dose, duration, and patient population most likely to benefit remain uncertain. A recent three-arm randomized clinical trial also suggested that zinc supplementation may enhance clinical improvement when combined with topical corticosteroids, whereas vitamin D did not clearly provide additional benefit over corticosteroid therapy alone [83].

The available evidence therefore does not support routine supplementation with antioxidants, vitamins, or micronutrients for all patients with OLP. Such interventions may be considered when a relevant nutritional deficiency is identified or when they are used as carefully selected adjunctive treatments. Importantly, biological plausibility or improvement in surrogate biomarkers should not be equated with established clinical efficacy.

## 6. Systemic Therapy for OLP

Systemic therapy is generally reserved for severe, extensive, rapidly progressive, or refractory OLP that cannot be adequately controlled with appropriate topical treatment. The evidence supporting systemic treatment is substantially weaker than that supporting topical corticosteroids, and treatment selection should take into account comorbidities, potential drug interactions, laboratory monitoring, and cumulative toxicity. Among systemic options, corticosteroids may be considered useful for short-term control of severe inflammatory disease, whereas systemic non-steroidal immunomodulators are generally reserved for selected refractory cases because of limited and heterogeneous evidence.

### 6.1. Systemic Corticosteroids

Systemic corticosteroids, particularly prednisolone, have been used for severe or refractory OLP. However, OLP-specific evidence remains limited and is largely derived from small clinical studies, with additional evidence extrapolated from lichen planus involving other sites [84,85,86,87]. Kuo et al. reported a high short-term response rate with locally injected triamcinolone combined with oral prednisolone in erosive OLP, but recurrence occurred during follow-up [86]. Lu et al. reported marked improvement with levamisole combined with low-dose prednisolone in refractory OLP [87].

*Overall evidence: Very low.*

*Clinical position: Reserved for severe, extensive, rapidly progressive, or refractory OLP when local treatment is insufficient.*

Systemic corticosteroids may provide relatively rapid suppression of severe inflammatory activity, but their benefit must be balanced against systemic adverse effects associated with prolonged exposure. They should therefore generally be used for limited periods and with careful monitoring rather than as long-term maintenance therapy.

### 6.2. Systemic Non-Steroidal Immunomodulators

Several systemic non-steroidal immunomodulators have been investigated for refractory OLP, including hydroxychloroquine, methotrexate, dapsone, levamisole, azathioprine, mycophenolate mofetil, colchicine, and other agents [88]. A scoping review identified 32 studies evaluating nine systemic non-steroidal immunomodulators and concluded that methotrexate and dapsone had the most supportive evidence among the available agents, while emphasizing the limited quality and heterogeneity of the evidence [88].

*Overall evidence: Low to very low, varying substantially by agent.*

*Clinical position: Selected treatment option for severe or refractory disease under specialist supervision.*

Hydroxychloroquine has shown clinical and symptomatic improvement in several studies, but a 2024 systematic review concluded that the available evidence remains insufficient to establish it as a routine treatment [89]. In a multicenter randomized controlled trial, hydroxychloroquine produced clinical improvement comparable to topical dexamethasone over 4 weeks but did not demonstrate superiority [90]. Thus, hydroxychloroquine may be considered in selected refractory cases, although current evidence is insufficient to support its routine use.

Methotrexate and dapsone may be promising options for refractory OLP, but the evidence remains insufficient to establish their relative superiority, optimal treatment duration, or long-term safety [88]. Evidence from lichen planus involving other sites also suggests potential efficacy of methotrexate, although its applicability to OLP remains uncertain [91]. Their use requires appropriate patient selection and monitoring for hematological, hepatic, renal, and other drug-specific adverse effects.

From an efficacy–safety perspective, systemic non-steroidal immunomodulators may offer an option for patients with refractory disease while avoiding prolonged systemic corticosteroid exposure; however, they introduce their own potential systemic toxicities and monitoring requirements. Therefore, the current evidence supports their use only as specialist-directed, individualized therapy rather than routine treatment.

### 6.3. Psychotropic Treatment

Psychotropic treatment has been investigated in selected patients with reticular OLP and burning-mouth-syndrome-like symptoms. A small pilot study suggested improvement in both oral and psychological symptoms in patients with refractory reticular OLP [92]. A four-case series also proposed a possible role for neurogenic inflammation and reported clinical improvement with adjunctive benzodiazepine therapy [93].

*Overall evidence: Very low.*

*Clinical position: Individualized adjunctive treatment for selected patients with persistent pain or prominent psychological or neuropathic symptoms.*

These findings are hypothesis-generating rather than confirmatory. Psychotropic agents should therefore not be regarded as disease-modifying treatment for OLP itself. Their potential role is more appropriately considered in patients in whom pain perception, anxiety, depressive symptoms, sleep disturbance, or burning-mouth-like symptoms contribute substantially to the clinical burden.

## 7. Overall Assessment of Efficacy and Safety

Taken together, the current evidence supports a pragmatic therapeutic hierarchy. Topical corticosteroids have the strongest clinical position and should remain the first-line therapy for symptomatic Oral lichen planus (OLP). TCIs, particularly tacrolimus and pimecrolimus, may be considered second-line options in selected patients, particularly those with inadequate response to topical corticosteroids. Laser-based interventions, herbal medicines, antioxidants, vitamins, and micronutrients may provide adjunctive benefits in selected circumstances, but the certainty of evidence remains low and their long-term efficacy and safety are insufficiently established [3,4,66,67,68,69,70,71,72,73,74,75,76,77,78,79,80,81,82,83,94].

Systemic corticosteroids remain useful for severe or refractory disease but should generally be limited because of systemic toxicity. Systemic non-steroidal immunomodulators, particularly methotrexate and dapsone, may be considered for carefully selected refractory cases, although evidence remains insufficient to define a preferred agent [88]. Hydroxychloroquine currently has insufficient evidence for routine use [88,90]. Psychotropic therapy should be reserved for selected patients with relevant comorbid psychological or neuropathic symptoms [92,93].

Thus, treatment selection should be based not simply on reported efficacy but on the balance between efficacy, certainty of evidence, adverse effects, treatment burden, comorbidities, and long-term safety. This approach is particularly important in OLP because treatment is often prolonged and complete cure is rarely achievable.

## 8. Malignant Transformation of Oral Lichen Planus

Oral lichen planus (OLP) was included among the oral potentially malignant disorders (OPMDs) in the 2017 WHO Classification of Head and Neck Tumours [95]. This designation has been retained in the 5th edition of the WHO Classification of Tumours, published in 2024, in which OLP remains included among OPMDs [96]. Although the malignant potential of OLP remains controversial, several studies have reported the development of oral squamous cell carcinoma (OSCC) in patients diagnosed with OLP [12,59,97]. A systematic review and meta-analysis by González-Moles and Ramos-García reported a pooled malignant transformation ratio of 1.43% (95% CI, 1.09–1.80%) for OLP, although substantial heterogeneity exists among studies because of differences in diagnostic criteria and study quality [5]. Earlier systematic reviews have reported lower overall estimates, including 1.09% in the systematic review by Fitzpatrick et al. [59]. However, the reported risk varies considerably among studies, partly because of differences in diagnostic criteria and the inclusion or exclusion of lesions with epithelial dysplasia or oral lichenoid lesions [5,58]. In particular, the significance and interpretation of epithelial dysplasia in OLP remain controversial, because inflammatory epithelial changes associated with OLP may overlap morphologically with dysplastic features [58]. Therefore, the malignant potential of OLP should be interpreted in the context of the diagnostic criteria and histopathological findings, and patients with OLP require appropriate long-term clinical follow-up.

Clinicians should remain vigilant for persistent or clinically changing lesions, particularly those showing persistent ulceration, progressive enlargement, induration, or other changes in surface characteristics. Red or erosive lesions and lesions involving the tongue may warrant particular attention because these clinical features have been associated with a higher risk of malignant transformation in OLP [5,59]. Persistent or clinically suspicious lesions, particularly those showing changes in morphology, size, surface characteristics, or consistency despite appropriate treatment, should be reassessed, and repeat biopsy should be considered to exclude epithelial dysplasia or OSCC [14,59]. Regular long-term follow-up is therefore essential, with particular attention to newly developed or changing lesions [14,59,97].

## 9. Conclusions and Future Directions

Oral lichen planus is a chronic, immune-mediated inflammatory disease with diverse clinical manifestations and an incompletely understood pathogenesis. Accurate diagnosis requires careful clinical and histopathological evaluation, particularly because OLP may overlap with oral lichenoid lesions and other oral mucosal disorders. Long-term management should focus on symptom control, suppression of clinically significant inflammation, maintenance of oral health, and elimination of potential precipitating or aggravating factors rather than on achieving a definitive cure.

Topical corticosteroids remain the preferred first-line pharmacological treatment for symptomatic OLP, while topical calcineurin inhibitors may be considered in selected patients, particularly those with corticosteroid-refractory disease. Laser therapy, herbal medicines, antioxidants, vitamins, micronutrients, and systemic immunomodulatory agents may have potential roles in selected patients, but the current evidence is generally limited by small sample sizes, methodological heterogeneity, and insufficient long-term follow-up. Systemic therapy should therefore be individualized according to disease severity, treatment response, comorbidities, and potential adverse effects.

Because OLP is recognized as an oral potentially malignant disorder, long-term clinical surveillance is an important component of management. Particular attention should be paid to newly developed or changing lesions, and repeat biopsy should be considered when clinical features raise concern for epithelial dysplasia or oral squamous cell carcinoma. Future research should focus on clarifying the molecular and immunological mechanisms underlying OLP, identifying clinically relevant biomarkers and therapeutic targets, and conducting well-designed, adequately powered clinical trials. Such efforts may contribute to more effective, evidence-based, and individualized approaches to the diagnosis and management of OLP.

## Figures and Tables

**Table 1:** Pathogenic and exacerbating factors associated with oral lichen planus.

① Pathogenic Factors	② Exacerbating Factors
Immune dysregulation T-cell-mediated immune responses and cytotoxic lymphocyte activity [13,16] Th17/Treg imbalance and IL-17–related inflammation [17,18] JAK/STAT signaling [21,22]	Lichenoid drug reactions Medications, including NSAIDs and antihypertensive agents, may trigger oral lichenoid reactions [35,40,41,42,43]
Epithelial barrier dysfunction Alterations in epithelial adhesion and tight-junction proteins [22,23]	Candida superinfection Candida infection may coexist with or exacerbate oral mucosal lesions, particularly during topical corticosteroid therapy [36,44]
Oral and gut microbiome alterations Dysbiosis of the oral microbiome [24,25] Gut microbiome dysbiosis and altered Treg regulation [20]	Poor oral hygiene and plaque accumulation Plaque control may improve clinical outcomes, particularly in gingival OLP [45,46]
Genetic susceptibility HLA-associated genetic susceptibility [28] Familial aggregation of lichen planus and OLP [29,30,31]	Xerostomia and altered salivary function Xerostomia and salivary gland hypofunction may contribute to oral discomfort and disease burden [37,39]
Infectious/systemic associations Association with hepatitis C virus infection, with considerable geographic and population variability [32,33,34]	Psychological factors Psychological distress and related factors may influence symptoms or disease course [38]
Multifactorial environmental/systemic influences Environmental and systemic factors may contribute to disease development and/or persistence [1,8,26,27]	Local oral conditions Local oral conditions and sources of irritation or inflammation may contribute to persistence or exacerbation of lesions [8,36,37,38,39,45,46]

Abbreviations: HLA, human leukocyte antigen; NSAIDs, nonsteroidal anti-inflammatory drugs; OLP, oral lichen planus; Treg, regulatory T cell.

## Data Availability

No new data were created or analyzed in this study.
